# Safety, Tolerability, and Pharmacokinetics of TAK-931, a Cell Division Cycle 7 Inhibitor, in Patients with Advanced Solid Tumors: A Phase I First-in-Human Study

**DOI:** 10.1158/2767-9764.CRC-22-0277

**Published:** 2022-11-14

**Authors:** Yasutoshi Kuboki, Toshio Shimizu, Kan Yonemori, Takashi Kojima, Shunsuke Kondo, Shigehiro Koganemaru, Satoru Iwasa, Kenichi Harano, Takafumi Koyama, Vickie Lu, Xiaofei Zhou, Huifeng Niu, Tomoko Yanai, Ignacio Garcia-Ribas, Toshihiko Doi, Noboru Yamamoto

**Affiliations:** 1 Department of Experimental Therapeutics, National Cancer Center Hospital East, Kashiwa, Japan.; 2 Department of Experimental Therapeutics, National Cancer Center Hospital, Tokyo, Japan.; 3 Quantitative Clinical Pharmacology, Takeda Development Center Americas, Inc. (TDCA), Lexington, Massachusetts, United States.; 4 Oncology Therapeutic Area Unit for Japan and Asia, Takeda Pharmaceutical Company Limited, Osaka, Japan.; 5 Oncology Early Development, Takeda Development Center Americas, Inc. (TDCA), Lexington, Massachusetts, United States.

## Abstract

**Purpose:**

We conducted a first-in-human, dose-escalation study, to evaluate the safety, tolerability, pharmacokinetics, pharmacodynamics, and activity of TAK-931, a cell division cycle 7 inhibitor, in Japanese patients with advanced solid tumors.

**Experimental Design:**

Patients ages ≥20 years received oral TAK-931: once daily for 14 days in 21-day cycles (schedule A; from 30 mg); once daily or twice daily for 7 days on, 7 days off in 28-day cycles (schedule B; from 60 mg); continuous once daily (schedule D; from 20 mg); or once daily for 2 days on, 5 days off (schedule E; from 100 mg) in 21-day cycles.

**Results:**

Of the 80 patients enrolled, all had prior systemic treatment and 86% had stage IV disease. In schedule A, 2 patients experienced dose-limiting toxicities (DLTs) of grade 4 neutropenia and the maximum tolerated dose (MTD) was 50 mg. In schedule B, 4 patients experienced DLTs of grade 3 febrile neutropenia (*n* = 3) or grade 4 neutropenia (*n* = 1); the MTD was 100 mg. Schedules D and E were discontinued before MTD determination. The most common adverse events were nausea (60%) and neutropenia (56%). Time to maximum plasma concentration of TAK-931 was approximately 1–4 hours postdose; systemic exposure was approximately dose proportional. Posttreatment pharmacodynamic effects correlating to drug exposure were observed. Overall, 5 patients achieved a partial response.

**Conclusions:**

TAK-931 was tolerable with a manageable safety profile. TAK-931 50 mg once daily days 1–14 in 21-day cycles was selected as a recommended phase II dose and achieved proof of mechanism.

**Trial registration ID:**

NCT02699749

**Significance:**

This was the first-in-human study of the CDC7 inhibitor, TAK-931, in patients with solid tumors. TAK-931 was generally tolerable with a manageable safety profile. The recommend phase II dose was determined to be TAK-931 50 mg administered once daily on days 1–14 of each 21-day cycle. A phase II study is ongoing to confirm the safety, tolerability, and antitumor activity of TAK-931 in patients with metastatic solid tumors.

## Introduction

Cell division cycle 7 (CDC7), a serine/threonine kinase (1), plays an important role in DNA replication (2). CDC7 drives G_1_–S-phase transition by binding to its regulatory protein, DBF4 (3). The CDC7–DBF4 complex, termed CDC7 kinase, initiates DNA replication via phosphorylation of a component of the minichromosome maintenance-2 helicase complex (MCM2) at Ser-40 (3, 4). It also regulates the DNA damage response (DDR) by modulating S-phase checkpoint signaling (5).

CDC7 overexpression may drive tumor proliferation in various malignancies (6–8), by suppressing DDR-mediated cell senescence and preventing DNA damage–induced apoptosis (9); overexpression has been associated with poor clinical outcomes (8, 10, 11) and may contribute to acquired resistance to chemotherapy (7, 8). Inhibition of CDC7 kinase by small-molecule inhibitors (12), anti-CDC7 antibody microinjection (13), or RNA silencing (14) induces antiproliferation in many cancer cell or immortal cell lines *in vitro.* Thus, CDC7 inhibitors have potential as novel cancer treatments.

TAK-931 (simurosertib) is an oral, highly potent, selective kinase inhibitor of CDC7 with demonstrated replication, stress-mediated antiproliferative activity across various cancer cell lines (15). In murine xenograft models of human colorectal, lung, ovarian, and pancreatic cancer, TAK-931 treatment causes significant and irreversible tumor growth inhibition, with favorable pharmacokinetic and pharmacodynamic profiles (15). In Japan, we conducted a first-in-human study of TAK-931 to evaluate the safety, tolerability, maximum tolerated dose (MTD), and recommended phase II dose (RP2D) in adults with advanced solid tumors.

## Materials and Methods

### Patients

Patients aged ≥20 years with histologically confirmed, advanced solid tumors (except primary brain tumors), an Eastern Cooperative Oncology Group performance status of 0/1, and for whom no effective standard therapy was available were eligible. Patients with seizures requiring antiepileptic treatment, symptomatic and/or progressive central nervous system metastases, blood pressure conditions, a history of ischemic myocardial, ischemic cerebrovascular, or thromboembolic events within 3 months before the first dose of TAK-931, or a history of orthostatic hypotension or syncope requiring medical intervention, or postural tachycardia syndrome were excluded. See Supplementary Materials and Methods for full eligibility criteria.

### Study Design

This was a phase I, open-label, dose-escalation study of single-agent TAK-931. Dose escalation of TAK-931 was cohort based with an adaptive design using Bayesian logistic regression modeling (BLRM) with pharmacokinetic guidance. BLRM also guided MTD estimation from the second dose. Patients were enrolled in one of four dosing schedules (A, B, D, or E; Supplementary Fig. S1) to receive TAK-931. Two additional schedules were planned (C and F) but no patients were enrolled. Schedule A included an initial accelerated escalation phase. TAK-931 was administered once daily for 14 days in 21-day cycles starting at 30 mg, which doubled until a cycle 1 dose-limiting toxicity (DLT) was observed, when 2 patients experienced grade ≥2 treatment-related toxicity, or when maximum geometric mean plasma concentration (*C*_max_) for the cohort reached or exceeded 800 ng/mL [threshold for off-target adverse effects (hypotension) in animal toxicology studies]; then dose escalation transitioned to modified Fibonacci escalation steps. In schedule B (based on experience with schedule A), patients received TAK-931 once daily or twice daily for 7 days on, 7 days off in a 28-day cycle starting at 60 mg. In schedule D, patients received TAK-931 once daily starting at 20 mg in 21-day cycles; BLRM was not applied but further adjustments were dependent on observed safety, pharmacokinetics, and pharmacodynamics. In schedule E, patients received TAK-931 once daily for 2 days on, 5 days off starting at 100 mg in 21-day cycles. A safety expansion was permitted once MTD had been determined. Prophylactic growth factors were permitted in all schedules to manage severe and/or febrile neutropenia. TAK-931 treatment continued until unacceptable toxicity, disease progression, or patient withdrawal.

The primary objectives were to evaluate safety, tolerability, and to identify the MTD of TAK-931. Secondary objectives were to characterize the pharmacokinetics of TAK-931, assess pharmacodynamic effects of TAK-931 by measuring basal and postdose levels of skin phosphorylated MCM2 (pMCM2; a CDC7 substrate), and assess preliminary clinical activity of TAK-931. An exploratory objective was to assess the pharmacodynamic effect of TAK-931 in fresh tumor biopsies after multiple doses of TAK-931 that were considered to have biological effect.

This study was conducted according to the protocol, the ethical principles that have their origin in the Declaration of Helsinki, the International Council for Harmonization Harmonized Tripartite Guideline for Good Clinical Practice, and all applicable regulations. The study protocol was reviewed and approved by the local or central Institutional Review Boards at all study sites. Patients provided written informed consent.

### Assessments

DLTs included: cycle 1 grade ≥3 hematologic and non-hematologic events or grade 2 non-hematologic events considered by the investigator to be TAK-931-related, grade 2 ejection fraction decrease, >2 weeks delay in initiating cycle 2 (1 week for schedules D and E), and >50% TAK-931 dose reduction in cycle 1 due to treatment-related adverse events (AEs; see Supplementary Materials and Methods for full DLT criteria). Toxicity was evaluated by NCI Common Terminology Criteria for Adverse Events (version 4.03). Tumor response was measured using the RECIST (version 1.1) (16). Serial blood samples for pharmacokinetic analysis were collected during cycle 1 on days 1 and 7 (schedule B), 1 and 8 (schedules A and D), or 1 and 9 (schedule E). Pharmacokinetic parameters were estimated from concentration–time profiles using noncompartmental methods with WinNonlin® Phoenix™ version 8.1 (Certara, Princeton, NJ). pMCM2 was detected semiquantitatively by IHC of histologic sections of formalin-fixed, paraffin-embedded skin and tumor biopsies using anti-pMCM2 (3378-1, Epitomics Inc.; ref. 15). Quantitative image analysis determined pMCM2 levels as “histologic score nuclei (H-score).” Skin punch biopsies (2–4 mm) were obtained during screening or predose on cycle 1, day 1 and for patients in schedules A, B, and D, postdose on any drug dosing day after 3 consecutive dosing days in cycle 1. A postdose skin biopsy was obtained on day 9 in schedule E. Fresh tumor biopsy pairs were collected predose and postdose on any dosing day after the completion of 3 consecutive dosing days in cycle 1 from patients who received doses considered to have biological effect.

### Statistical Analysis

The safety population included patients who received ≥1 dose of TAK-931. The pharmacodynamic-evaluable population comprised patients from the safety population with a baseline and ≥1 additional postbaseline biopsy sample that was suitable for pMCM2 analysis. The pharmacokinetic-evaluable population included patients with sufficient dosing and TAK-931 concentration–time data to estimate pharmacokinetic parameters. The response-evaluable population included patients in the safety population with measurable disease at baseline and ≥1 postbaseline response assessment.

### Data Availability Statement

The datasets, including the redacted study protocol, redacted statistical analysis plan, and individual participants’ data supporting the results reported in this article, will be made available within 3 months from initial request, to researchers who provide a methodologically sound proposal. The data will be provided after its deidentification, in compliance with applicable privacy laws, data protection, and requirements for consent and anonymization.

## Results

### Patients

The safety population comprised 80 patients (enrolled and treated between March 24, 2016 and December 12, 2019) who received TAK-931 at the following doses: 30, 40, 60 (*n* = 3 each), and 50 mg (*n* = 16) in schedule A; 60 mg (*n* = 3), 80 mg (*n* = 9), 100 mg (*n* = 6), and 120 mg (*n* = 6) in schedule B; 20 mg, 30 mg, and 40 mg (*n* = 6 each) in schedule D, and 100 mg (*n* = 4), 120 mg (*n* = 3), and 150 mg (*n* = 6) in schedule E.

Patient demographics and baseline characteristics are shown in Table 1. Median age was 59 years overall. Common diagnoses included esophageal squamous cell cancer (16%) and pancreatic cancer (13%). Patients had advanced disease (86% stage IV) and were heavily pretreated. At data cutoff (April 6, 2020), all patients had discontinued the study, mostly due to progressive disease (90%; Supplementary Fig. S2).

**TABLE 1:** Patient baseline demographics and disease characteristics

	Schedule A *n* = 25	Schedule B *n* = 24	Schedule D *n* = 18	Schedule E *n* = 13	Total *N* = 80
Male, *n* (%)	15 (60)	15 (63)	10 (56)	8 (62)	48 (60)
Median age, years (range)	59 (42–75)	58 (36–75)	61.5 (36–78)	62 (45–75)	59 (36–78)
Cancer type, *n* (%)^a^					
Bile duct	0	2 (8)	0	0	2 (3)
Bladder	2 (8)	0	0	0	2 (3)
Unknown primary origin	1 (4)	2 (8)	0	0	3 (4)
Cervical	2 (8)	0	2 (11)	0	4 (5)
Colon	0	1 (4)	1 (6)	1 (8)	3 (4)
Colorectal	1 (4)	0	1 (6)	1 (8)	3 (4)
Esophageal	1 (4)	3 (13)	6 (33)	3 (23)	13 (16)
Gall bladder	1 (4)	1 (4)	0	0	2 (3)
Gastric	2 (8)	0	1 (6)	0	3 (4)
Ovarian	1 (4)	2 (8)	0	0	3 (4)
Pancreatic	4 (16)	3 (13)	3 (17)	0	10 (13)
Prostate	0	0	0	2 (15)	2 (3)
Rectal	2 (8)	0	2 (11)	0	4 (5)
Sarcoma	1 (4)	2 (8)	0	0	3 (4)
Disease stage at study entry (%)					
III	1 (4)	0	0	0	1 (1)
IIIB	1 (4)	0	0	0	1 (1)
IV	21 (84)	19 (79)	17 (94)	12 (92)	69 (86)
IVB	1 (4)	1 (4)	1 (6)	1 (8)	4 (5)
Not available	1 (4)	4 (17)	0	0	5 (6)
ECOG performance status, *n* (%)					
0	19 (76)	16 (67)	16 (89)	8 (62)	59 (74)
1	6 (24)	8 (33)	2 (11)	5 (38)	21 (26)
Prior systemic lines of therapy, median (range)	4 (1–10)	3 (1–10)	3 (1–12)	4 (2–18)	3.0 (1–18)

Abbreviation: ECOG, Eastern Cooperative Oncology Group.

^a^Diagnoses in 1 patient only—schedule A: bile duct carcinoma, bile tract cancer, breast, duodenal cancer, paranglioma, peritoneal carcinoma, thymic carcinoma (squamous cell); schedule B: allantoic duct cancer, anal cancer, kidney cancer, mesothelioma, non–small cell lung cancer, rectal carcinoids, thymic carcinoma nos, thymus cancer; schedule D: duodenum papilla cancer, melanoma; schedule E: cecal cancer, duodenal papilla cancer, duodenum cancer, penile cancer, thymic carcinoma, thymus cancer.

### DLTs and MTD

In schedule A, 2 of 3 patients receiving TAK-931 60 mg experienced DLTs of grade 4 neutropenia lasting >3 days, per the protocol definition of DLT at the time; the MTD was determined as 50 mg based on DLT incidence, relative dose intensity, pharmacokinetic, and AE profiles at this dose. In schedule B, DLTs of grade 3 febrile neutropenia were experienced by 2 of 9 patients receiving TAK-931 80 mg and one of six patients receiving TAK-931 120 mg. One of 6 patients receiving TAK-931 100 mg experienced a DLT of grade 4 neutropenia lasting >7 days (following a protocol amendment, the criteria for grade 4 neutropenia being considered a DLT was changed from lasting >3 days to lasting >7 days). The MTD for this schedule was determined as 100 mg based on DLTs, a low relative dose intensity, and high frequency of AEs at 120 mg. Although DLTs were reported in schedules D (1 patient experienced grade 4 neutropenia at 40 mg) and E (1 patient with grade 4 neutropenia and a second patient with grade 3 febrile neutropenia, both received 150 mg), MTDs were not determined as dose escalation was discontinued.

### Safety and Tolerability

Across all schedules, patients received a median of 3.0 (range, 1–21) cycles of TAK-931; most (64%) received ≥3 cumulative cycles. Mean treatment duration was 86.3 days (range, 4–469). Median TAK-931 relative dose intensity across all schedules was 95.0% (range, 29%–100%). AEs were experienced by 79 patients (99%), most commonly nausea (60%), neutropenia (56%), vomiting (36%), white blood cell count decreased (34%), alopecia (33%), decreased appetite (29%), anemia (24%), and leukopenia (24%; Table 2). Cardiovascular events occurring in ≥10% of patients overall were hypotension (14%), electrocardiogram QT prolonged, and orthostatic hypotension (5% each), all grade 1/2. Grade ≥3 AEs were reported by 50 patients (63%) and included neutropenia (46%), white blood cell count decreased (15%), leukopenia (14%), anemia (13%), and febrile neutropenia (6%; Table 3). Twenty-three patients (29%) experienced ≥1 serious AE, including febrile neutropenia [*n* = 4 (5%)], malignant ascites, and decreased appetite [*n* = 3 (4%) each].

**TABLE 2:** Most common AEs (reported in ≥10% patients in any schedule)

Preferred term, *n* (%)	Schedule A *n* = 25	Schedule B *n* = 24	Schedule D *n* = 18	Schedule E *n* = 13	Total *N* = 80
Non-hematologic					
Nausea	15 (60)	21 (88)	5 (28)	7 (54)	48 (60)
Vomiting	7 (28)	13 (54)	2 (11)	7 (54)	29 (36)
Alopecia	6 (24)	13 (54)	3 (17)	4 (31)	26 (33)
Decreased appetite	7 (28)	5 (21)	6 (33)	5 (38)	23 (29)
Diarrhea	7 (28)	4 (17)	1 (6)	3 (23)	15 (19)
Fatigue	2 (8)	5 (21)	3 (17)	2 (15)	12 (15)
Malaise	5 (20)	1 (4)	3 (17)	2 (15)	11 (14)
Hypotension	2 (8)	4 (17)	0 (0)	5 (38)	11 (14)
Constipation	2 (8)	5 (21)	2 (11)	1 (8)	10 (13)
Edema peripheral	4 (16)	1 (4)	2 (11)	3 (23)	10 (13)
Pyrexia	5 (20)	1 (4)	2 (11)	2 (15)	10 (13)
Tumor pain	3 (12)	1 (4)	1 (6)	3 (23)	8 (10)
Blood creatinine increased	3 (12)	3 (13)	1 (6)	0 (0)	7 (9)
Stomatitis	0 (0)	6 (25)	0 (0)	1 (8)	7 (9)
Arthralgia	3 (12)	1 (4)	1 (6)	1 (8)	6 (8)
Dry skin	1 (4)	1 (4)	1 (6)	3 (23)	6 (8)
Nasopharyngitis	2 (8)	3 (13)	1 (6)	0 (0)	6 (8)
Abdominal pain	2 (8)	3 (13)	0 (0)	0 (0)	5 (6)
Gamma-glutamyltransferase increased	0 (0)	3 (13)	0 (0)	2 (15)	5 (6)
Electrocardiogram QT prolonged	3 (12)	0 (0)	0 (0)	1 (8)	4 (5)
Hypoalbuminemia	4 (16)	2 (8)	0 (0)	0 (0)	6 (8)
Pruritus	0 (0)	3 (13)	1 (6)	0 (0)	4 (5)
Orthostatic hypotension	0 (0)	1 (4)	0 (0)	3 (23)	4 (5)
Fall	0 (0)	1 (4)	1 (6)	2 (15)	4 (5)
Rash popular	0 (0)	3 (13)	2 (11)	0 (0)	5 (6)
Cough	3 (12)	0 (0)	0 (0)	0 (0)	3 (4)
Hematologic					
Neutropenia	12 (48)	19 (79)	6 (33)	8 (62)	45 (56)
White blood cell count decreased	8 (32)	5 (21)	7 (39)	7 (54)	27 (34)
Anemia	6 (24)	4 (17)	3 (17)	6 (46)	19 (24)
Leukopenia	5 (20)	13 (54)	0 (0)	1 (8)	19 (24)
Platelet count decreased	2 (8)	1 (4)	2 (11)	1 (8)	6 (8)
Thrombocytopenia	1 (4)	4 (17)	0 (0)	0 (0)	5 (6)
Febrile neutropenia	1 (4)	3 (13)	0 (0)	1 (8)	5 (6)
Lymphopenia	2 (8)	3 (13)	0 (0)	0 (0)	5 (6)
Lymphocyte count decreased	3 (12)	0 (0)	0 (0)	1 (8)	4 (5)

**TABLE 3:** Most common grade ≥3 AEs (reported in >1 patient in any schedule)

Preferred term, *n* %	Schedule A *n* = 25	Schedule B *n* = 24	Schedule D *n* = 18	Schedule E *n* = 13	Total *N* = 80
Non-hematologic					
Decreased appetite	2 (8)	0 (0)	1 (6)	0 (0)	3 (4)
Gamma-glutamyltransferase increased	0 (0)	2 (8)	0 (0)	0 (0)	2 (3)
Hypoalbuminemia	0 (0)	2 (8)	0 (0)	0 (0)	2 (3)
Hypophosphatemia	1 (4)	1 (4)	0 (0)	0 (0)	2 (3)
Hematologic					
Neutropenia	11 (44)	15 (63)	3 (17)	8 (62)	37 (46)
White blood cell count decreased	3 (12)	3 (13)	2 (11)	4 (31)	12 (15)
Leukopenia	5 (20)	6 (25)	0 (0)	0 (0)	11 (14)
Anemia	1 (4)	3 (13)	1 (6)	5 (38)	10 (13)
Febrile neutropenia	1 (4)	3 (13)	0 (0)	1 (8)	5 (6)
Lymphocyte count decreased	1 (4)	0 (0)	0 (0)	1 (8)	2 (3)

Forty-nine patients (61%) experienced AEs resulting in TAK-931 dose modification, including dose reductions (31%), dose holds (45%), or dose delays (40%). Four patients (5%) discontinued treatment: 3 due to pleural effusion, abdominal abscess, and anemia (*n* = 1 each), and 1 due to febrile neutropenia, pneumonia aspiration, and sepsis. Two patients (3%), with pancreatic and thymic cancer, respectively, died during the study due to disease progression; neither were related to TAK-931.

### Pharmacokinetics

**FIGURE 1:**
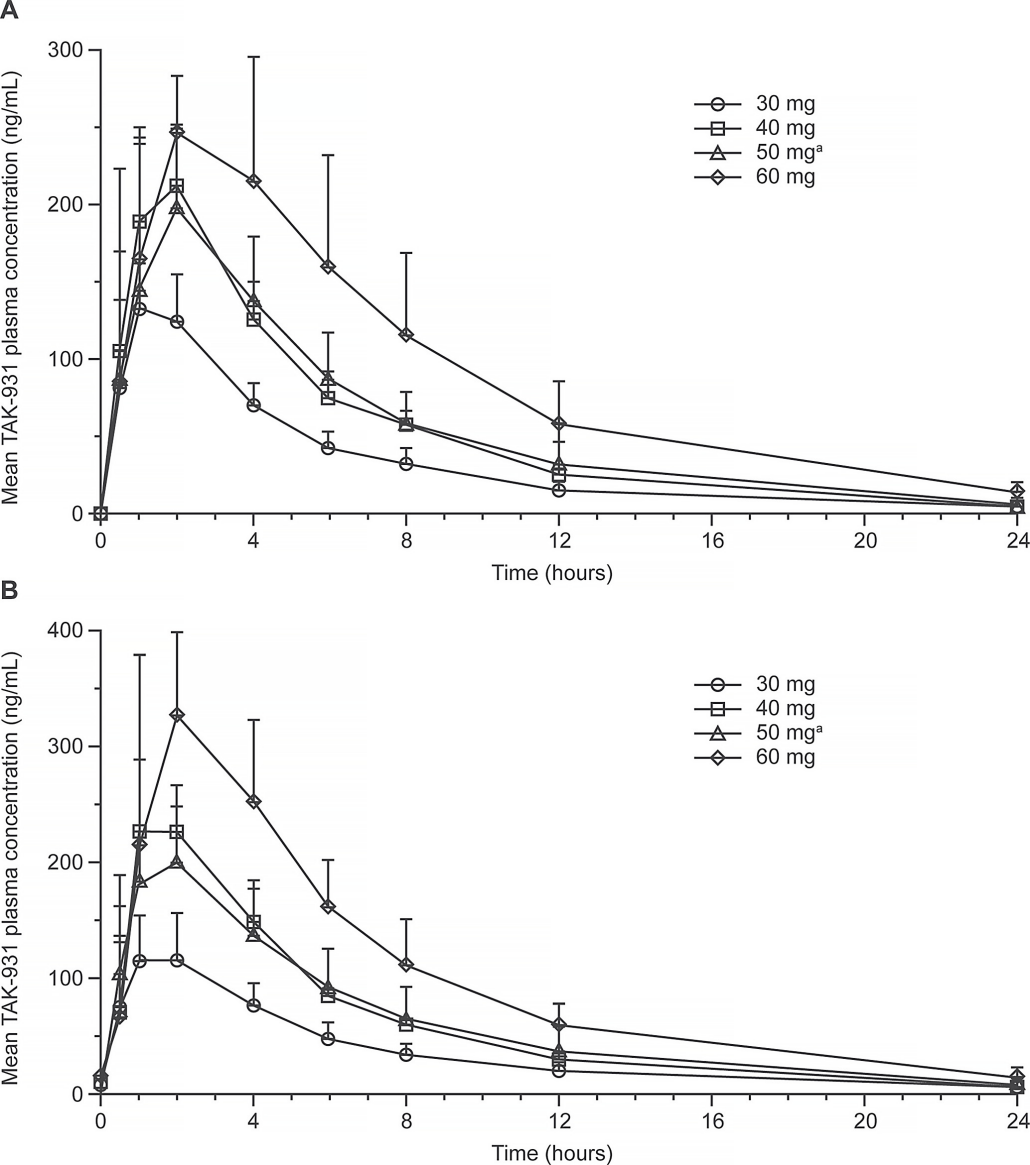
Mean (+StDev) plasma concentration–time profiles of TAK-931 in patients from schedule A after: a single dose (**A**) or multiple doses (**B**) of TAK-931 30–60 mg at cycle 1, day 1 in the pharmacokinetic-evaluable population (linear scale). The number of patients included in each dose were *n* = 3 at 30 mg, *n* = 3 at 40 mg, *n* = 15 (single dose)/*n* = 14 (multiple doses) at 50 mg, *n* = 3 at 60 mg. ^a^Concentration data from 1 patient in the 50 mg cohort were excluded. StDev, standard deviation.

TAK-931 concentration–time data were obtained from all patients. Mean plasma concentration–time profiles after single and multiple dosing for schedule A are shown in Fig. 1. Mean plasma concentration–time profiles for schedules B, D, and E are shown in Supplementary Fig. S3. Median time to maximum plasma concentration (*T*_max_) was achieved approximately 1–4 hours postdose over the dose range of 20–50 mg, and approximately 2 hours postdose with the 50 mg dose. On the basis of dose-normalized area under the concentration–time curve (0–24 hours; AUC_24_), TAK-931 systemic exposure was approximately dose proportional following single- and multiple-dose administration over the range 20–150 mg. Mean half-life (*T*_1/2_) of TAK-931 following oral TAK-931 administration was 4.58–6.53 hours. Systemic exposures of TAK-931 remained mostly unchanged between day 1 and days 7, 8, or 9, with minor accumulation following higher doses (120 and 150 mg). The overall mean accumulation ratio (*R*_ac[_*_C_*_max]_ and *R*_ac[AUC]_) of 0.789 to 1.920 was consistent with the estimated *T*_1/2_ of approximately 5–6 hours. On the basis of the estimated amount of TAK-931 excreted in urine, renal clearance (CLr) of TAK-931 was approximately 8% of total apparent clearance (*n* = 79).

### Pharmacodynamics

**FIGURE 2:**
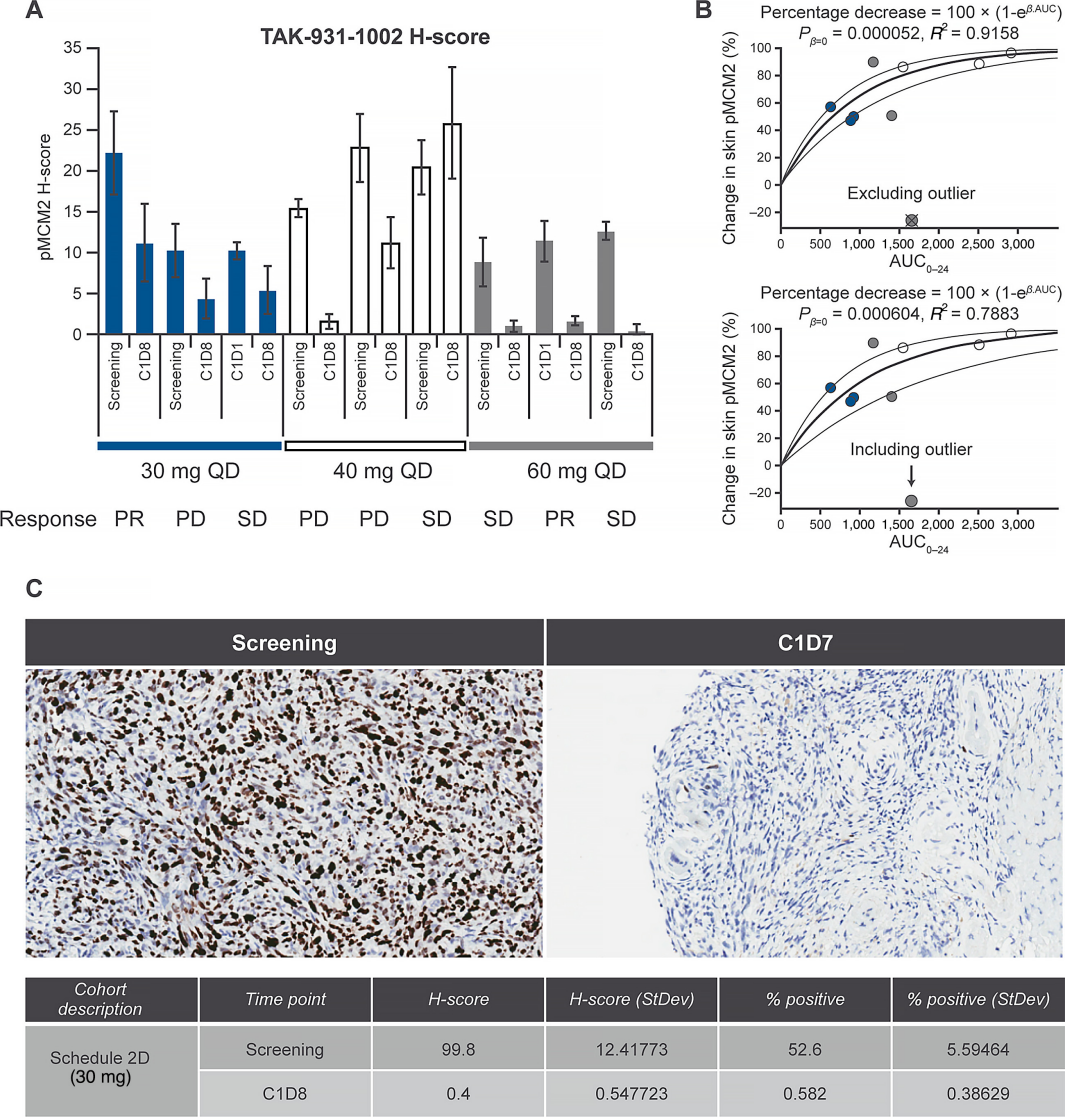
TAK-931 pharmacodynamic effect. pMCM2 H-scores in skin biopsies from 9 individual patients at screening or at cycle 1, day 1 and post-TAK-931 treatment at cycle 1, day 8 (**A**); correlation of percentage decrease in skin pMCM2 with exposure to TAK-931 (_0–24_) in patients in schedule A (**B**); and inhibition of pMCM2 in a tumor biopsy from a single patient with melanoma in schedule D who had a best response of SD (**C**). AUC, area under the plasma concentration–time curve; AUC_0–24_, area under the plasma concentration-time curve from the time 0 to 24 hours; C, cycle; D, day; PD, progressive disease, PR, partial response, SD, stable disease, StDev, standard deviation.

Pharmacodynamic response to TAK-931 was assessed in skin biopsies from 47 patients predose and on cycle 1, day 8 postdose (Table 4). TAK-931 suppressed skin pMCM2 levels from 34.1% to 90.9% postdose. Individual H-scores from baseline and postdose as well as best overall response of 9 patients with evaluable skin biopsy data in schedule A are shown in Fig. 2A. Tumor type and best response to TAK-931 for these patients are also shown in Supplementary Table S1. Suppression of skin pMCM2 by TAK-931 appeared to be dose dependent and was positively correlated with TAK-931 exposure (AUC_0–24;_*P*_β = 0_ = 0.000052, *R*^2^ = 0.9158; Fig. 2B). TAK-931 also suppressed pMCM2 in tumor tissue biopsies as shown by IHC staining for pMCM2 in an individual patient from schedule D dosed at 30 mg (Fig. 2C).

**TABLE 4:** Pharmacodynamic effect of TAK-931 on pMCM2 expression in skin punch biopsies measured as a percent change from baseline of the mean H-score (during screening or predose on cycle 1, day 1 and cycle 1, day 8)

		Baseline	Cycle 1, day 8	% of pMCM2 inhibition
	*n*	Mean H-score (StDev)	Mean H-score (StDev)	(Change from baseline)
Schedule A (30 mg)	3	18.47 (8.45)	10.93 (6.57)	40.8%
Schedule A (40 mg)	3	19.53 (3.78)	12.87 (12.19)	34.1%
Schedule A (50 mg)	14	10.50 (5.85)	3.76 (5.42)	64.2%
Schedule A (60 mg)	3	10.93 (1.94)	1.00 (0.60)	90.9%
Schedule B (60 mg)	3	4.40 (2.96)	0.67 (0.46)	84.6%
Schedule B (80 mg)	9	12.72 (2.02)	3.54 (6.19)	72.2%
Schedule D (20 mg)	6/4^a^	22.05 (24.52)	5.05 (2.90)	77.1%
Schedule D (30 mg)	6	33.32 (23.43)	6.43 (5.17)	80.7%

Abbreviation: StDev, standard deviation.

^a^Baseline: *n* = 6; cycle 1, day 8: *n* = 4.

### Antitumor Activity

Seventy-five patients were response evaluable (Supplementary Table S2). In schedule A, overall response rate (ORR) was 13%: 1 patient with duodenal squamous cell cancer receiving TAK-931 30 mg achieved a partial response (PR) maintained for 2.1 months; 1 patient with cervical squamous cell cancer receiving TAK-931 60 mg achieved a PR maintained for 2.2 months, and 1 patient with esophageal squamous cell cancer receiving TAK-931 50 mg achieved a PR maintained for 2.7 months. Overall, diagnoses of esophageal, thymic, or cervical cancer accounted for 39% of patients with stable disease (SD) or achieving PR. One patient with adenosquamous type duodenal cancer and metastasis site with a squamous component achieved PR. Change from baseline in tumor size for patients in schedule A is shown in Supplementary Fig. S4A and computerized tomography scans from 2 responding patients are shown in Supplementary Fig. S4B and S4C. In schedule B, ORR was 9%: 1 patient with esophageal cancer receiving TAK-931 100 mg achieved a PR maintained for 4.2 months and 1 patient with anal cancer receiving TAK-931 120 mg achieved a PR but discontinued at the same visit on the advice of the investigator due to worsening of symptoms (hoarseness and dysphagia). Overall, 28 patients achieved a best response of SD. One patient with cervical cancer in schedule D (40 mg) achieved SD lasting for 15.24 months. Two patients in schedule A (50 mg) with cervical cancer and thymic cancer achieved SD for 11.33 and 10.45 months, respectively. Overall, median progression-free survival was 2.2 months (95% confidence interval, 1.94–3.06).

## Discussion

This was the first-in-human study of a CDC7 inhibitor in patients with solid tumors. The MTD of TAK-931 was 50 mg once daily on days 1–14 of a 21-day treatment cycle (schedule A), or 100 mg on days 1–7 and days 15–21 of a 28-day cycle (schedule B) based on DLTs, relative dose intensity, and AE profiles. Dose escalation of continuous daily dosing and twice-weekly dosing schedules were discontinued because of changes in the sponsor's development strategy and not as a result of toxicity or lack of efficacy; thus, no MTD was established. Due in part to the lack of indication that higher TAK-931 doses resulted in greater antitumor activity, the sponsor decided against expanding the MTD of schedule B; therefore, the MTD of schedule A was evaluated as the RP2D.

Treatment with TAK-931 was generally tolerable with an acceptable safety profile in this population. All DLTs were grade 4 neutropenia or grade 3 febrile neutropenia. The most common AEs were gastrointestinal or hematologic in nature. Most AEs were grade 1/2, excluding neutropenia, which was the most frequently reported grade ≥3 AE (46%). Overall, AEs were manageable with dose modifications; 4 patients discontinued treatment, and patients remained on treatment for an average of 86.3 days. The frequency of hematologic and gastrointestinal AEs appeared higher with schedule B compared with schedule A. TAK-931 50 mg once daily on days 1–14 of a 21-day cycle (schedule A) was selected for further development in a phase II study (NCT03261947).

TAK-931 was rapidly absorbed (*T*_max_ ∼2 hours) following oral administration of 50 mg, with a relatively short-terminal half-life (∼5 hours), supporting daily dosing. Plasma exposure was approximately dose proportional following single- and multiple-dose administration over 20–50 mg. These results are consistent with a previous population pharmacokinetic analysis of TAK-931 (17).

Analysis of the pharmacokinetic-pharmacodynamic relationship using data from xenograft models determined that TAK-931 inhibition of pMCM2 in tumor correlated well with inhibition in skin (Takeda, data on file), and established pMCM2 inhibition in skin as a potential surrogate biomarker for pMCM2 inhibition in tumor cells, although confirmatory studies are needed. Here, TAK-931, at the dose and schedule selected for a phase II investigation, led to strong, dose-dependent inhibition of skin and tumor pMCM2, which correlated with TAK-931 exposure. These data demonstrated target engagement of TAK-931 through inhibition of phosphorylation of the direct downstream target of CDC7, providing clinical evidence of the predicted mechanism of action for TAK-931. Future studies investigating how pMCM2 inhibition in skin relates to the clinical antitumor effect could be of interest.

TAK-931 showed activity in patients with advanced-stage, largely chemoresistant disease. Three (13%) patients in schedule A and 2 (9%) patients in schedule B achieved a PR. Diagnoses of esophageal cancer, cervical cancer, and thymic cancer, which typically affect the squamous cell tissue, accounted for 39% of patients achieving a best response of PR or SD. CDC7 overexpression has been observed after oncogenic transformation in squamous cell tissue (7, 8); however, baseline screening for CDC7 overexpression in tumors was not conducted. One patient with adenosquamous type duodenal cancer and a metastasis site with a squamous component had a best response of PR. CDC7 overexpression has also been implicated in chemoresistance development in esophageal cancer, with sensitivity restored via CDC7 knockout (7). These observations suggest that squamous cell carcinomas may be sensitive to treatment with TAK-931; however, larger studies are required to confirm.

There are other CDC7 inhibitors in development, some of which are undergoing clinical investigation. XL413 (BMS-863233) demonstrated potent CDC7-dependent cell-cycle arrest and *in vivo* tumor growth inhibition in preclinical studies; however, both clinical trials that were initiated have since been terminated (NCT00838890 NCT00886782; ref. 18). TQB3824 is a small-molecule CDC7 inhibitor that demonstrated antitumor efficacy in solid tumor models with CDC7 overexpression; it is being investigated in a phase I trial enrolling patients with advanced cancer (NCT05028218; ref. 19). LY3143921 is an orally administered ATP-competitive inhibitor of CDC7 and in a phase I clinical study of patients with advanced solid tumors enriched for malignancies associated with TP53 mutations (NCT03096054), LY3143921 was well tolerated but demonstrated limited single-agent clinical activity suggesting that further analyses would need to investigate rational combination approaches (20). Interestingly, this study also excluded patients with significant baseline hypotension, and reported rates of drug-related orthostatic hypotension of 50%, suggesting that hypotension may be a class effect of CDC7 inhibition (20). In our study, cardiovascular (hypotension) and renal toxicity were anticipated on the basis of preclinical animal studies (Takeda, data on file); however, the eligibility criteria excluded patients with a history of cardiovascular and blood pressure conditions to mitigate potential toxicity, and clinical and urine chemistry were used to monitor potential renal effects. Cardiovascular events were all grade 1/2 and nonserious and no clinically relevant decrease in CLr was observed.

TAK-931 has potential applications in combination with other DDR inhibitors, including poly ADP ribose polymerase (PARP) inhibitors. PARP inhibitors are used to treat malignancies that exhibit the “BRCAness” phenotype, which is the loss of function of the homologous recombination DDR pathway (21). Impairment of two DDR mechanisms can prevent one pathway compensating for the loss of the other (22). Preclinical findings suggest that TAK-931 could induce “BRCAness” in cancer cells, enhancing antiproliferative activity of PARP inhibitors (23). Indeed, TAK-931 suppressed DNA repair activity and enhanced the biological activity of PARP inhibitors, topoisomerase inhibitors, and platinum compounds in human xenograft models (23, 24). While there is potential for overlapping hematologic toxicity, clinical investigation of these combinations is warranted.

## Conclusions

TAK-931 was tolerable, with a manageable safety profile in patients with advanced solid tumors. On the basis of these results, TAK-931 50 mg once daily on days 1–14 of a 21-day cycle is being investigated in a phase II study in patients with metastatic pancreatic cancer, colorectal cancer, esophageal squamous cell cancer, or squamous non–small cell lung cancer (NCT03261947). A tablet formulation of TAK-931 is in development to address scalability in larger trials (NCT03708211).

## Supplementary Material

Supplementary Methods SM1 Supplementary methods including inclusion and exclusion criteria

Table ST1 Summary of best response to treatment for patients in the pharmacodynamic-evaluable population (schedule A).

Table ST2 Summary of best response to treatment per investigator’s assessment.

Figure SF1 Dosing, PK, and pharmacodynamic sampling schedules.

Figure SF2 Patient disposition.

Figure SF3 Mean (+StDev) plasma concentration-time profiles of TAK-931 in patients after: (A) a single dose or (B) multiple doses of TAK-931 at cycle 1, day 1 by schedule (linear scale) in the pharmacokinetic-evaluable population.

Figure SF4 (A) Best percent change from baseline in tumor size for all patients in schedule A. (B) and (C) computed tomography scans from patients in schedule A at screening (left panels) and 9 weeks post-dose (right panels); (B) patient with duodenal cancer treated with TAK-931 30 mg; (C) patient with esophageal cancer treated with TAK-931 50 mg. Yellow arrows indicate target lesions.
