# Supplementary material for: Safety, Tolerability, and Pharmacokinetics of TAK-931, a Cell Division Cycle 7 Inhibitor, in Patients with Advanced Solid Tumors: A Phase I First-in-Human Study
Source: Cancer Res Commun. 2022 Nov 14;2(11):1426–35. doi: 10.1158/2767-9764.CRC-22-0277 (PMC10035389; doi:10.1158/2767-9764.CRC-22-0277)
Supplement: Supplementary Methods SM1 — Supplementary methods including inclusion and exclusion criteria [file crc-22-0277-s01.docx]

**Supplementary Data (Online Only)**

**Article Type:** Research Article

**Title: Safety, Tolerability, and Pharmacokinetics of TAK-931, a Cell Division Cycle 7 Inhibitor, in Patients with Advanced Solid Tumors: A Phase 1 First-in-Human Study**

**Authors:** Yasutoshi Kuboki, Toshio Shimizu, Kan Yonemori, Takashi Kojima, Shunsuke Kondo, Shigehiro Koganemaru, Satoru Iwasa, Kenichi Harano, Takafumi Koyama, Vickie Lu, Xiaofei Zhou, Huifeng Niu, Tomoko Yanai, Ignacio Garcia-Ribas, Toshihiko Doi, and Noboru Yamamoto

**Supplementary Methods**

**Inclusion Criteria**

Each patient met all of the following inclusion criteria to be enrolled in the study:

1. Male or female patients ≥20 years old

2. Histologically confirmed diagnosis of an advanced, non-hematologic (solid) tumor (with the exception of primary brain tumor)

3. Eastern Cooperative Oncology Group performance status of 0 or 1

4. Patients for whom no effective standard therapy was available

5. Life expectancy of ≥3 months

6. Female patients who:

- Were postmenopausal (natural amenorrhea and not due to other medical reasons) for ≥1 year before the screening visit, or
- Were surgically sterile, or
- If they were of childbearing potential, agreed to practice two effective methods of contraception, at the same time, from the time of signing the informed consent through 30 days after the last dose of study drug, or
- Agreed to practice true abstinence, when this was in line with the preferred and usual lifestyle of the patient (periodic abstinence [e.g., calendar, ovulation, symptothermal, post-ovulation methods] and withdrawal were not acceptable methods of contraception)

Male patients, even if surgically sterilized (e.g., status post-vasectomy), who:

- Agreed to practice effective barrier contraception during the entire study treatment period and through 120 days after the last dose of study drug, or
- Agreed to practice true abstinence, when this was in line with the preferred and usual lifestyle of the patient (periodic abstinence [e.g., calendar, ovulation, symptothermal, post-ovulation methods for the female partner] and withdrawal were not acceptable methods of contraception)
- Agreed not to donate sperm during this study and for 120 days after receiving their last dose of study drug

7. Voluntary written consent was given before performance of any study-related procedure not part of standard medical care, with the understanding that consent may be withdrawn by the patient at any time without prejudice to future medical care

8. Ability to swallow oral medications, willingness to undergo serial skin punch biopsies, and suitable venous access for the study-required pharmacokinetic and pharmacodynamic sampling

9. Clinical laboratory values as specified below within 28 days before the first dose of study drug:

- Bone marrow reserve consistent with an absolute neutrophil count (ANC) ≥1500/mm^3^, platelet count ≥100,000/mm^3^, and hemoglobin ≥9 g/dL
- Total bilirubin was <1.5 times the upper limit of normal (ULN)
- Alanine aminotransferase or aspartate aminotransferase was ≤3 × ULN. Aspartate aminotransferase and alanine aminotransferase may have been elevated up to 5 × ULN if the elevation could be reasonably ascribed to the presence of hepatocellular carcinoma, biliary tract cancer, or metastatic disease in the liver
- Serum albumin ≥3.0 g/dL
- Serum creatinine <1.5 × ULN or creatinine clearance based on the Cockcroft-Gault estimate ≥50 mL/minute for patients with serum creatinine concentrations above institutional limits

10. Left ventricular ejection fraction >50% as measured by echocardiogram or multigated acquisition scan (MUGA) within 4 weeks before receiving the first dose of study drug

11. Recovered (grade ≤1 toxicity) from the reversible effects of prior anticancer therapy. Patients with ongoing toxicities at baseline were eligible; however, any grade 2 baseline toxicity (except for alopecia) was discussed with the medical monitor

**Exclusion Criteria**

Patients meeting any of the following exclusion criteria were not enrolled in the study:

1. Patients who required continuous use of proton pump inhibitors or histamine-2 receptor antagonists, and patients who were taking proton pump inhibitors within 5 days before the first dose of study drug

2. Treatment with clinically significant enzyme inducers within 14 days before the first dose of study drug

3. Treatment with any investigational products within 30 days before the first dose of study drug

4. Female patients who were lactating and breastfeeding or had a positive serum pregnancy test during the screening period or a positive urine pregnancy test on day 1 before the first dose of study drug

5. Any serious medical or psychiatric illness that could, in the investigator’s opinion, potentially have interfered with the completion of treatment according to this protocol

6. History of any of the following within the last 3 months before administration of the first dose of study drug:

- Ischemic myocardial event including angina requiring therapy and artery revascularization procedures, myocardial infarction, and unstable symptomatic ischemic heart disease
- Ischemic cerebrovascular event, including transient ischemic attack and artery revascularization procedures
- Thromboembolic events (e.g., deep vein thrombosis, pulmonary embolism, or symptomatic cerebrovascular events)
- Significant, uncontrolled cardiac arrhythmia (including atrial flutter/fibrillation, ventricular fibrillation, or ventricular tachycardia)
- Use of rate control drugs for arrhythmias (including beta blockers [such as metoprolol], acetylcholine, digoxin, and non-dihydropyridine calcium channel blockers diltiazem and verapamil)
- Placement of a pacemaker for control of cardiac rhythm
- Requirement for inotropic support (including digoxin)
- New York Heart Association class II to IV heart failure
- Any other cardiac condition that in the opinion of the investigator could pose an additional risk for the participation in the study (e.g., pericardial effusion or restrictive cardiomyopathy)
- Baseline prolongation of the rate-corrected QT interval (QTc, e.g., repeated demonstration of a QTc interval >480 msec, or a history of congenital, long QT syndrome, or torsades de pointes)

7. Patients with any of the following blood pressure conditions:

- History of orthostatic hypotension or syncope that required medical intervention. Orthostatic hypotension was defined as a 20 mmHg fall in systolic blood pressure and/or a 10 mmHg fall in diastolic blood pressure within 2 to 5 minutes of quiet standing immediately after a 5-minute period of supine rest
- Postural orthostatic tachycardia syndrome or postural tachycardia syndrome (defined as an increase in heart rate of >30 beats per minute over baseline after 10 minutes of quiet standing)
- Hypertension that was unstable or not controlled by medication

8. Seizures requiring antiepileptic treatment

9. History of uncontrolled brain metastasis unless:

- Previously treated with surgery, whole-brain radiation, or stereotactic radiosurgery
- Stable disease for ≥60 days, without steroid use (or stable steroid dose established for ≥28 days before the first dose of TAK-931)

10. Symptomatic and/or progressive central nervous system metastases

11. Ongoing medical conditions, such as acute exacerbations of chronic illnesses, serious infections, or major surgery within 4 weeks before receiving the first dose of study drug

12. Known history of HIV infection

13. Known hepatitis B surface antigen seropositive or detectable hepatitis C infection viral load. Note: Patients who had positive hepatitis B core antibody or hepatitis B surface antibody could be enrolled but must have had an undetectable hepatitis B viral load. Patients who had positive hepatitis C antibody must have had an undetectable hepatitis C viral load

14. Known gastrointestinal (GI) disease or GI procedure that could have interfered with the GI absorption of study drug, such as total gastrectomy or GI conditions that could have substantially modified gastric pH

**Criteria for Dose-Limiting Toxicities**

A dose-limiting toxicity (DLT) was defined as any of the following events that occurred during cycle 1 and were considered by the investigator to be related to TAK-931:

- Grade 4 neutropenia (ANC <500 cells/mm^3^) lasting >7 consecutive days; if myeloid growth factors were used, the event was considered a DLT irrespective of the duration
- Grade ≥3 neutropenia (ANC <1000 cells/mm^3^) with fever and/or infection, where fever was defined as a single temperature of >38.3°C or sustained temperature of ≥38°C for >1 hour
- Grade 4 thrombocytopenia lasting >7 consecutive days; a platelet count of <10,000/mm^3^ at any time was considered a DLT
- Grade ≥3 thrombocytopenia of any duration accompanied by grade 2 bleeding or requiring transfusion
- Delay in the initiation of cycle 2 by >14 days (7 days for schedules D, E, and F) due to a lack of adequate recovery of treatment-related hematologic or non-hematologic toxicities
- Grade 2 ejection fraction decreased by echocardiogram or multigated acquisition scan
- Other grade 2 non-hematologic toxicities considered by the investigator to be related to the study drug and dose-limiting
- Patients who received <50% of doses of the planned TAK-931 dosing in cycle 1 for related adverse events: <7 once-daily (QD) doses or <14 twice-daily (BID) doses for schedules A and B; <4 QD doses or <7 BID doses for schedule C and; <11 QD doses or <21 BID doses for schedule D, <3 QD doses or <6 BID doses for schedule E, or <2 QD doses or <3 BID doses for schedule F
- Grade ≥3 non-hematologic toxicity with the following exceptions:
  - Grade 3 arthralgia/myalgia that responded to nonsteroidal anti-inflammatory drugs
  - Grade 3 fatigue lasting <1 week
- Isolated grade ≥3 laboratory abnormalities if it was asymptomatic and resolved to grade ≤1 or baseline levels in ≤7 days
- Grade 3 nausea and/or emesis that could be controlled to grade <3 in ≤3 days with the use of optimal antiemetics (defined as an anti-emetic regimen that employed both a 5-hydroxytryptamine [serotonin] type 3 receptor antagonist and a corticosteroid given in standard doses and according to standard schedules)
- Grade 3 diarrhea that could be controlled to grade <3 in ≤3 days with appropriate treatment
