# Supplementary material for: Safety, Tolerability, and Pharmacokinetics of TAK-931, a Cell Division Cycle 7 Inhibitor, in Patients with Advanced Solid Tumors: A Phase I First-in-Human Study
Source: Cancer Res Commun. 2022 Nov 14;2(11):1426–35. doi: 10.1158/2767-9764.CRC-22-0277 (PMC10035389; doi:10.1158/2767-9764.CRC-22-0277)
Supplement: Table ST1 — Summary of best response to treatment for patients in the pharmacodynamic-evaluable population (schedule A). [file crc-22-0277-s02.docx]

**Supplementary Table S1.** Summary of best response to treatment for patients in the pharmacodynamic-evaluable population (schedule A).

|  | **Cancer type** | **Best response** |
| --- | --- | --- |
| TAK-931 30 mg | Pancreatic | SD |
|  | Duodenal | PR |
|  | Rectal | PD |
| TAK-931 40 mg | Breast | PD |
|  | Unknown | SD |
|  | Rectal | PD |
| TAK-931 60 mg | Ovarian | SD |
|  | Cervical | PR |
|  | Pancreatic | SD |

Abbreviations: PD, progressive disease; PR, partial response; SD, stable disease.
