# Supplementary material for: Safety, Tolerability, and Pharmacokinetics of TAK-931, a Cell Division Cycle 7 Inhibitor, in Patients with Advanced Solid Tumors: A Phase I First-in-Human Study
Source: Cancer Res Commun. 2022 Nov 14;2(11):1426–35. doi: 10.1158/2767-9764.CRC-22-0277 (PMC10035389; doi:10.1158/2767-9764.CRC-22-0277)
Supplement: Table ST2 — Summary of best response to treatment per investigator’s assessment. [file crc-22-0277-s03.docx]

**Supplementary Table S2.** Summary of best response to treatment per investigator’s assessment.

| **Response *n* (%) [95% CI]** | **Schedule A**  ***n* = 24** | **Schedule B**  ***n* = 23** | **Schedule D**  ***n* = 17** | **Schedule E**  ***n* = 11** |
| --- | --- | --- | --- | --- |
| ORR | 3 (13) [3–32] | 2 (9) [1–28] | 0 | 0 |
| CR | 0 | 0 | 0 | 0 |
| PR | 3 (13) [3–32] | 2 (9) [1–28] | 0 | 0 |
| SD | 9 (38) [19–59] | 10 (43) [23–66] | 5 (29) [10–56] | 4 (36) [11–69] |
| PD | 12 (50) [29–71] | 11 (48) [27–69] | 12 (71) [44–90] | 7 (64) [31–89] |

Abbreviations: CI, confidence interval; CR, complete response; PD, progressive disease; PR, partial response; ORR, overall response rate; SD, stable disease.
