# Supplementary material for: Safety, Tolerability, and Pharmacokinetics of TAK-931, a Cell Division Cycle 7 Inhibitor, in Patients with Advanced Solid Tumors: A Phase I First-in-Human Study
Source: Cancer Res Commun. 2022 Nov 14;2(11):1426–35. doi: 10.1158/2767-9764.CRC-22-0277 (PMC10035389; doi:10.1158/2767-9764.CRC-22-0277)
Supplement: Figure SF1 — Dosing, PK, and pharmacodynamic sampling schedules. [file crc-22-0277-s04.docx]

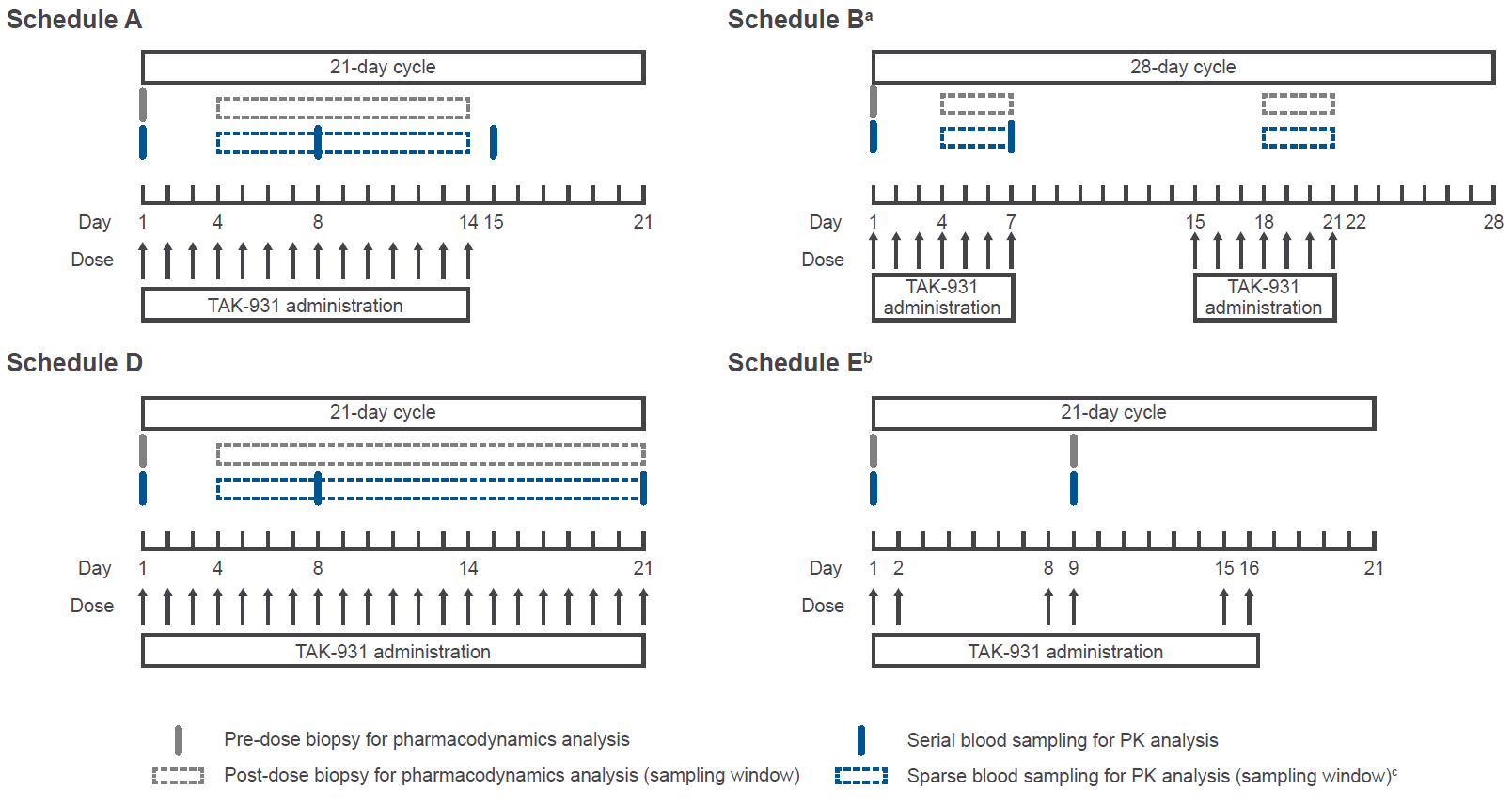


**Supplementary Figure S1.**

Dosing, PK, and pharmacodynamic sampling schedules. ^a^In the case of unacceptable adverse events leading to dosing delays, this schedule could be modified to: 7 days on, 14 days off in a 21-day cycle (schedule C). ^b^If safety data suggested that a once-weekly schedule would allow for a higher dose escalation, this schedule could be modified to: 1 day on, 6 days off (schedule F). ^c^If the skin or tumor biopsy was taken on a nonseries PK sampling day, sparse PK plasma sample was collected at pre-dose, at 1–3 hours post-dose, and at 4–9 hours post-dose on the day of biopsy. PK, pharmacokinetic
