# Supplementary figures and images for: Safety, Tolerability, and Pharmacokinetics of TAK-931, a Cell Division Cycle 7 Inhibitor, in Patients with Advanced Solid Tumors: A Phase I First-in-Human Study
Source: Cancer Res Commun. 2022 Nov 14;2(11):1426–35. doi: 10.1158/2767-9764.CRC-22-0277 (PMC10035389; doi:10.1158/2767-9764.CRC-22-0277)

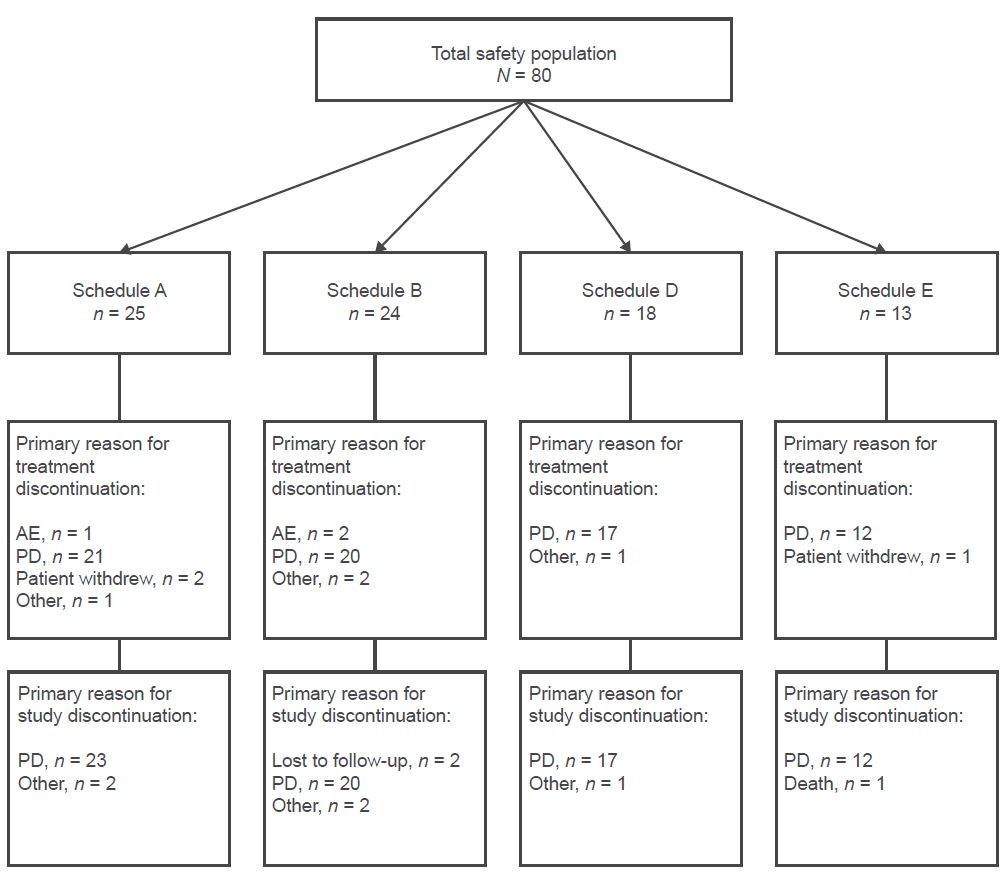


**Supplementary Figure S2.**

Patient disposition. AE, adverse event; PD, progressive disease.

Supplement: Figure SF2 — Patient disposition. [file crc-22-0277-s05.docx]
