# Supplementary material for: Safety, Tolerability, and Pharmacokinetics of TAK-931, a Cell Division Cycle 7 Inhibitor, in Patients with Advanced Solid Tumors: A Phase I First-in-Human Study
Source: Cancer Res Commun. 2022 Nov 14;2(11):1426–35. doi: 10.1158/2767-9764.CRC-22-0277 (PMC10035389; doi:10.1158/2767-9764.CRC-22-0277)
Supplement: Figure SF3 — Mean (+StDev) plasma concentration-time profiles of TAK-931 in patients after: (A) a single dose or (B) multiple doses of TAK-931 at cycle 1, day 1 by schedule (linear scale) in the pharmacokinetic-evaluable population. [file crc-22-0277-s06.docx]

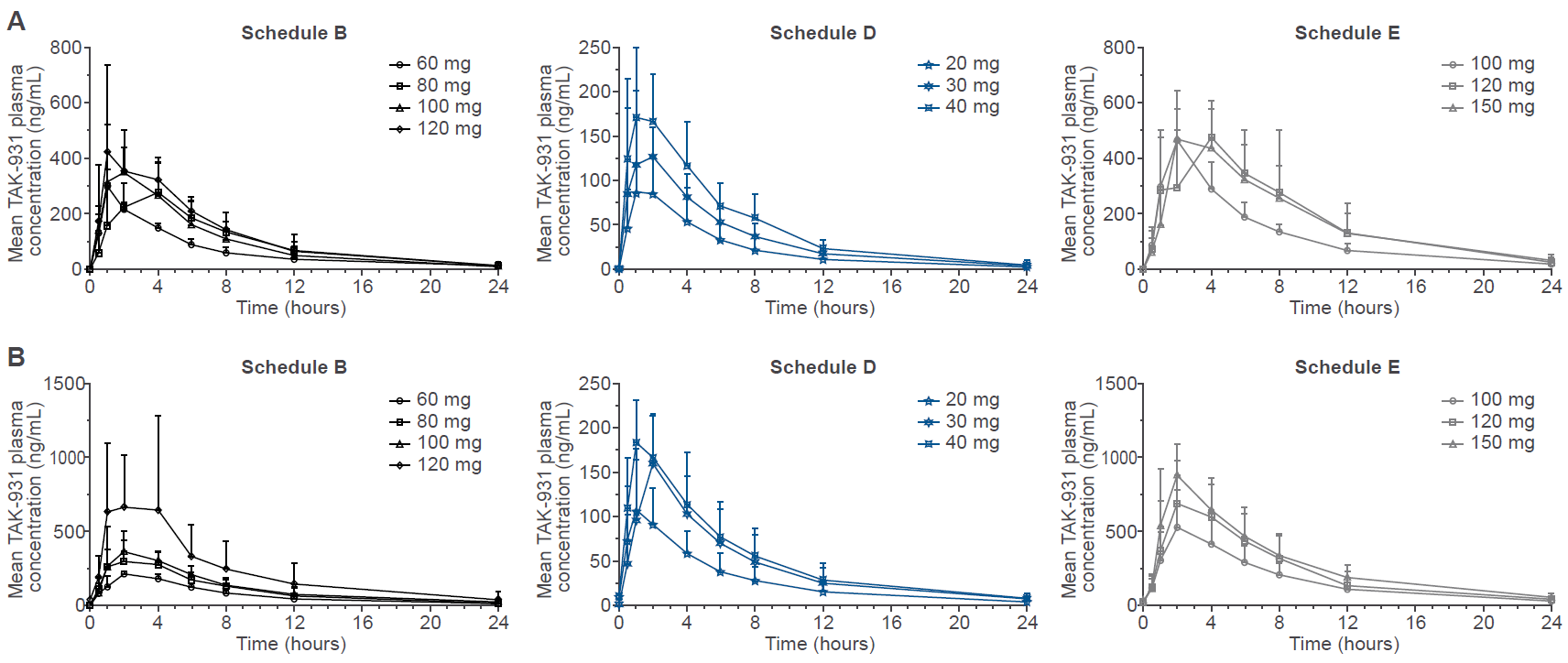


**Supplementary Figure S3.**

Mean (+StDev) plasma concentration-time profiles of TAK-931 in patients after: (**A**) a single dose or (**B**) multiple doses of TAK-931 at cycle 1, day 1 by schedule (linear scale) in the pharmacokinetic-evaluable population. Schedule B: TAK-931 60, 80, 100, or 120 mg once daily or twice daily on days 1–7 and 14–21 in 28-day cycles. Schedule D: TAK-931 20, 30, or 40 mg once daily on days 1–21 in 21-day cycles. Schedule E: TAK-931 100, 120, or 150 mg once daily on days 1–2, 7–9, and 14–16 in 21-day cycles. StDev, standard deviation
