# Supplementary material for: Safety, Tolerability, and Pharmacokinetics of TAK-931, a Cell Division Cycle 7 Inhibitor, in Patients with Advanced Solid Tumors: A Phase I First-in-Human Study
Source: Cancer Res Commun. 2022 Nov 14;2(11):1426–35. doi: 10.1158/2767-9764.CRC-22-0277 (PMC10035389; doi:10.1158/2767-9764.CRC-22-0277)
Supplement: Figure SF4 — (A) Best percent change from baseline in tumor size for all patients in schedule A. (B) and (C) computed tomography scans from patients in schedule A at screening (left panels) and 9 weeks post-dose (right panels); (B) patient with duodenal cancer treated with TAK-931 30 mg; (C) patient with esophageal cancer treated with TAK-931 50 mg. Yellow arrows indicate target lesions. [file crc-22-0277-s07.docx]

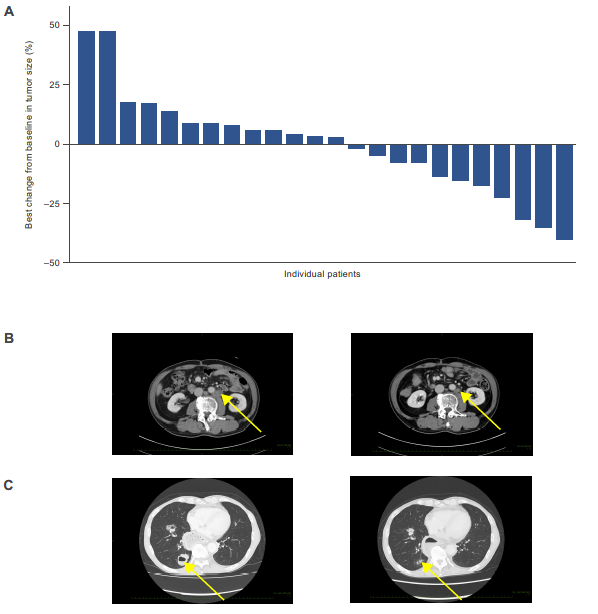


**Supplementary Figure S4.**

(**A**) Best percent change from baseline in tumor size for all patients in schedule A. (**B**) and (**C**) computed tomography scans from patients in schedule A at screening (left panels) and 9 weeks post-dose (right panels); (**B**) patient with duodenal cancer treated with TAK-931 30 mg; (**C**) patient with esophageal cancer treated with TAK-931 50 mg. Yellow arrows indicate target lesions.
